# Trends in non-communicable disease mortality among adult residents in Nairobi's slums, 2003–2011: applying InterVA-4 to verbal autopsy data

**DOI:** 10.3402/gha.v7.25533

**Published:** 2014-10-29

**Authors:** Samuel O. Oti, Steven van de Vijver, Catherine Kyobutungi

**Affiliations:** 1 African Population and Health Research Center, Nairobi, Kenya; 2 Department of Global Health, Academic Medical Centre, University of Amsterdam, Amsterdam, The Netherlands; 3 Amsterdam Institute for Global Health and Development, Amsterdam, The Netherlands; 4 INDEPTH Network, Accra, Ghana

**Keywords:** cause of death, verbal autopsy, interval, non-communicable diseases, mortality, Kenya, sub-Saharan Africa

## Abstract

**Background:**

About 80% of deaths from non-communicable diseases (NCDs) occur in developing countries such as Kenya. However, not much is known about the burden of NCDs in slums, which account for about 60% of the residences of the urban population in Kenya. This study examines trends in NCD mortality from two slum settings in Nairobi.

**Design:**

We use verbal autopsy data on 1954 deaths among adults aged 35 years and older who were registered in the Nairobi Urban Health and Demographic Surveillance System between 2003 and 2011. InterVA-4, a computer-based program, was used to assign causes of death for each case. Results are presented as annualized cause-specific mortality rates (CSMRs) and cause-specific mortality fractions (CSMFs) by sex.

**Results:**

The CSMRs for NCDs did not appear to change significantly over time for both males and females. Among males, cardiovascular diseases (CVDs) and neoplasms were the leading NCDs – contributing CSMFs of 8 and 5%, respectively, on average over time. Among females, CVDs contributed a CSMF of 14% on average over time, while neoplasms contributed 8%. Communicable diseases and related conditions remained the leading causes of death, contributing a CSMF of over 50% on average in males and females over time.

**Conclusions:**

Our findings are consistent with the Global Burden of Disease 2010 study which shows that communicable diseases remain the dominant cause of death in Africa, although NCDs were still significant contributors to mortality. We recommend an integrated approach towards disease prevention that focuses on health systems strengthening in resource-limited settings such as slums.

Non-communicable diseases (NCDs) are traditionally considered to be diseases of affluent societies. However, global projections show that the largest increase in deaths from NCDs will occur in low-resource settings where, already, 80% of global NCD deaths occur (1, 2). Specifically, the evidence indicates that four major NCDs – cardiovascular disease (CVD), cancer, diabetes, and chronic respiratory disease – together make the largest contribution to the burden of NCDs globally and in low- and middle-income countries (LMICs) (2). These trends have been strongly linked with high rates of urbanization and changes in individual and societal lifestyle in LMICs, such as an increase in tobacco use, excessive alcohol consumption, reduced physical activity, and adoption of ‘Western’ diets that are high in salt, refined sugar, and unhealthy fats and oils (3). Population ageing is also believed to be associated with increased risk of NCDs in LMICs (4).

The rising burden of NCDs is of particular concern among poor communities partly because of lack of awareness and limited access to quality health care. The poor are often affected disproportionately by NCDs due to having only limited access to preventive and curative services, and having to spend a higher proportion of their income on health care costs for these lifelong conditions. This has the potential to further widen health inequalities within and between countries (5). The urban poor, resident in vast slums across many LMIC cities (6), are most vulnerable to NCDs. This is because of high exposure to risk factors like poor diets, excessive alcohol consumption, tobacco use, stress and environmental pollution, and limited access to screening, preventive, and curative services for NCDs (7–10). Specifically, there is some evidence that the levels of risk factors for common NCDs like smoking and reduced physical activity are relatively higher in the slums in LMICs compared to the rural areas (10). Considering that the majority of urban populations in LMICs reside in slums or slum-like conditions (6, 11, 12), the potential impact of an NCD epidemic on these populations cannot be ignored. Unfortunately, the public health systems in most LMICs are not prepared to deal with the rising burden of NCDs, even though cost-effective interventions for NCD prevention and control are described (13–15). In order to ensure that NCDs receive attention in the allocation of limited resources for health care provision in most LMICs, it is important to have robust evidence on the burden of NCDs in such settings.

One way to quantify the burden of the NCDs is through population-level data on mortality attributable to NCDs. Unfortunately, most of what is known about NCDs in LMICs is based on statistical estimates since most of these countries lack the complete vital registration–type data needed to generate actual population-level figures such as national NCD mortality estimates (16–18). Statistical estimates of mortality such as those based largely on hospital records may be inaccurate as they will fail to capture deaths outside a health facility – which account for up to 60% of deaths in a country like Kenya (19). In the absence of vital registration systems and data on causes of death, Health and Demographic Surveillance Systems (HDSS) provide a useful platform for contributing to the understanding of the population-level mortality. Typically, HDSS monitor and track demographic and health indicators in a population within a defined geographic area (20). HDSS also typically generate cause-of-death data using a process known as *verbal autopsy* (VA) – in which retrospective interviews are conducted with primary caregivers of deceased persons to determine the circumstances surrounding deaths (21). These interviews can be analysed to determine the most likely causes of death in the population under surveillance to inform health planning (22).

This article is based on cause-of-death data generated from VA in two slums covered by the Nairobi Urban Health and Demographic Surveillance System (NUHDSS) in Kenya. Specifically, we examine trends in NCD mortality in the NUHDSS from 2003 to 2011 – the earliest and latest years, respectively, for which VA data are complete.

## Methods

### Study area and population

We used VA data from deaths that occurred among adults aged 35 years and older in the NUHDSS between 2003 and 2011. Details about the operation of the NUHDSS have been described elsewhere (23). In brief, the NUHDSS covers the two slums of Viwandani and Korogocho, both located less than 10 km from Nairobi, the capital of Kenya. In total, the population under surveillance in both slums is about 72,000 people, living in close to 28,500 households. We focus on deaths in adults aged 35 years and older due to the fact that the largest proportion of deaths due to NCDs is typically expected in this age group, and based on our analysis (results not shown) of younger age groups which showed very few NCD deaths. Moreover, slum populations like ours tend to have fewer older people compared to the INDEPTH 2013 standard (24). The data used in this analysis can be accessed via the INDEPTH Network Data Repository (25).

### Cause-of-death data in the NUHDSS

Cause-of-death data using VA are generated in the NUHDSS for the two slum areas under surveillance, and full details of this process have also been published elsewhere (26, 27). In brief, trained interviewers visit a household, on average within 3 months after a death has occurred, to conduct a VA interview with a credible respondent – usually a spouse or other close family member who would be aware of the circumstances surrounding the death. The interview is conducted using a semi-structured questionnaire that mostly inquires about probable symptoms and signs that the deceased may have portrayed before death.

### Interpretation of VA data – the InterVA model

In order to generate causes of death, the data from completed VA questionnaires are entered into InterVA-4 – a computer program that applies probabilistic modelling to arrive at possible causes of death. We use InterVA because it is a freely accessible (open source) and widely validated program for cause-of-death interpretation. Full details of the InterVA model, including its use in interpreting cause-of-death data from HDSS across the developing world, have also been described in previous publications (28–38). Briefly, the data in the completed VA questionnaires were captured electronically into an SQL database, converted into a comma-separated value (.csv) file, and imported into STATA 12 (StataCorp. 2011. *Stata Statistical Software: Release 12*. College Station, TX: StataCorp LP). Using a STATA script, the data in the .csv file were converted into the input indicators required by the InterVA probabilistic model. The resultant input indicator file, also in .csv format, was run in InterVA-4's batch mode which in turn generates likely causes of death. A maximum of three likely causes of death per case with a likelihood value between 0 and 1 was produced by InterVA-4 for each cause. If the likelihood cause-of-death values for a particular death did not sum to 1, the difference between the sum of the likelihood values for probable causes of death and 1 were allocated to the ‘indeterminate’ cause. As recommended in the InterVA-4 user guide (39), all identified causes were considered proportionate to their likelihood values in the mortality rate calculations In other words, to get the total number of deaths due to each cause, we sum the three likelihood values generated by InterVA and then divide this by the person years (PYs) (see below) to arrive at the cause-specific mortality rate (CSMR). The output from InterVA-4 was then imported into STATA for analysis.

### Statistical approach

All analysis was performed using STATA 13 (StataCorp. 2013. *Stata Statistical Software: Release 13*. College Station, TX: StataCorp LP). Deaths and observed PYs were aggregated annually for all individuals in the study population for the period from 1 January 2003 to 31 December 2011. Individuals contributed PYs as long as they were living in the NUHDSS area. Residents stopped contributing time if they out-migrated and resumed contributing if they re-entered the NUHDSS area. We classified cause of death by three main groups: Group I – communicable, maternal, perinatal, and nutritional conditions; Group II – NCDs; and Group III – injuries, according to the Global Burden of Disease classification (40). NCD-related deaths are further sub-categorized into two major groups – neoplasms and CVDs. Due to few numbers, all other NCDs are classified as ‘Other NCD’. Those cases for which no cause of death could be determined by InterVA-4 were classified as ‘Indeterminate’. Annual mortality rates, expressed as deaths per 10,000 PYs, were then calculated for each of the above cause-of-death categories by gender and age group. Results are presented as annualized group- and/or CSMRs and cause-specific mortality fractions (CSMFs) by sex and age group. We then apply the Mann–Kendall statistical test (41, 42) to the annualized group-specific mortality data. This test is a non-parametric test for identifying trends in time series data. One benefit of this test is that the data need not conform to any particular distribution. That is, no assumption of normality is required. Typically, the test is used to determine whether the central or median value of the random response variable of interest changes over time. We report the results of the Mann–Kendall statistic, S, for each annualized group-specific mortality trend line. Let *x*_1_, *x*_2_, … *x*_*n*_ represent *n* data points where *x*_j_ represents the data point at time *j*. Then, the basic specification for S is given by:

$$S=\sum_{k=1}^{n-1}\sum_{j=k+1}^{n}sign(x_{j}-x_{k})$$

where

$$\begin{array}{c} sign\left(x_{j}-x_{k}\right)=1\text{ if}\left(x_{j}-x_{k}\right)>0\\ =0\text{ if}\left(x_{j}-x_{k}\right)=0\\ =-1\text{ if}\left(x_{j}-x_{k}\right)<0 \end{array}$$

The variance of S is calculated and used to compute a normalized test statistic, *Z*_S_. Finally, the probability of *Z*_S_ is computed at a 95% significance level. The trend is said to be decreasing if *Z*_S_ is negative and the computed probability is greater than the level of significance. The trend is said to be increasing if the *Z*_S_ is positive and the computed probability is greater than the level of significance. If the computed probability is less than the level of significance, there is no trend.

### Ethical considerations

We utilized data that are routinely collected by the NUHDSS. In order to operate the NUHDSS, APHRC applied for and received approval from the Kenya Medical Research Institution's Ethics Review Committee (KEMRI/ERC).

## Results

**Fig. 1:**
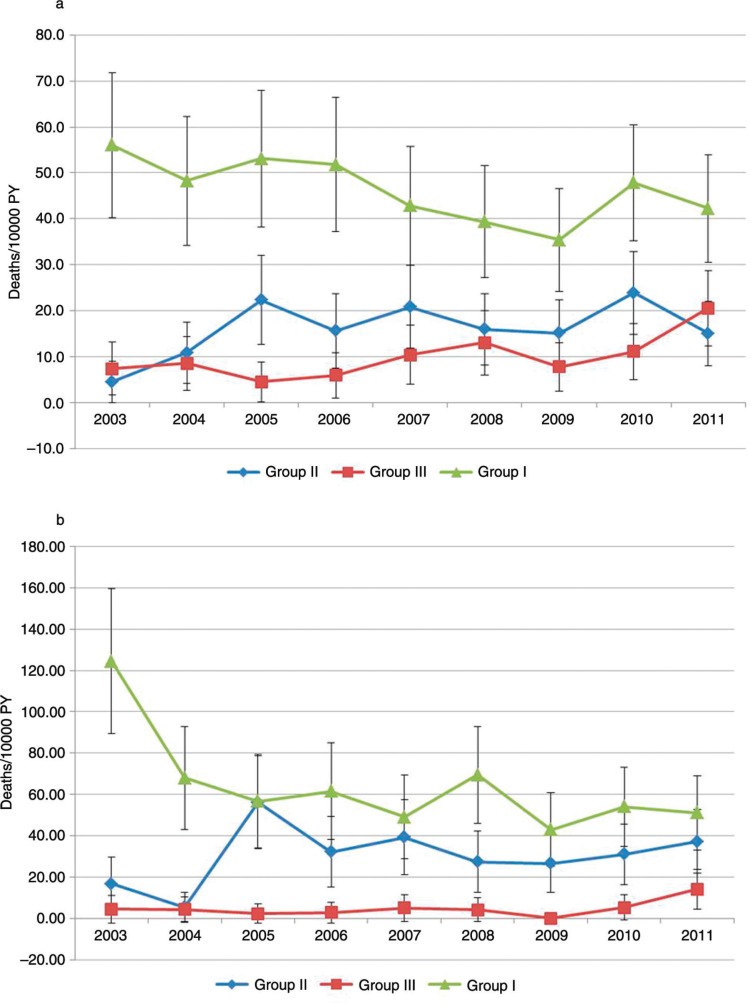
Trends in cause-specific mortality rates among adults aged 35 years and older by major cause-of-death groups by year per 10,000 person years, 2003–2011: (a) males; and (b) females. Group I – Communicable, maternal, perinatal, and nutritional conditions; Group II – non-communicable diseases; and Group III – injuries.

Between 1 January 2003 and 31 December 2011, there were 1954 deaths among the 132,973 PYs observed in adults aged 35 years and older in the NUHDSS. Of these deaths, 1,217 (62%) occurred in men and 737 (38%) occurred in females. Table 1 shows the age–gender distribution of deaths by year. Figure 1 shows trends in the CSMRs per 10,000 PYs by major cause-of-death group among males and females, respectively. Among males, NCDs (group II) show the second highest levels of CSMRs (after group I – communicable, maternal, perinatal, and nutritional conditions) over the entire study period. However, the trend in CSMRs for NCDs over the study period does not show any significant upward or downward trajectory, with values fluctuating between 4.5 and 23 deaths per 10,000 PYs at various non-regular intervals over the study period. This is confirmed by a non-significant normalized Mann–Kendall statistic (*Z*_S_=0.73). Overall, group I showed the highest overall CSMR, whereas group III (comprising injuries) showed the lowest levels of CSMR over time – with the former showing a significant downward trend (*Z*_S_=−2.19) and the latter showing a significant upward trend (*Z*_S_=1.98) over time.

**Table 1:** Age–gender distribution of deaths by year among adults aged 35 years and older, 2003–2011

	Males (*N*=1,217)		Females (*N*=737)	
		
	<50 years	50+ years	<50 years	50+ years
Year	*n* (%)	*n* (%)	*n* (%)	*n* (%)
2003	80 (67)	39 (33)	75 (68)	35 (32)
2004	86 (64)	49 (36)	48 (66)	25 (34)
2005	69 (59)	47 (41)	31 (44)	40 (56)
2006	65 (53)	57 (47)	43 (65)	23 (35)
2007	81 (57)	61 (43)	30 (43)	40 (57)
2008	82 (65)	45 (35)	51 (73)	19 (27)
2009	63 (62)	39 (38)	33 (55)	28 (46)
2010	111 (63)	66 (37)	55 (52)	51 (48)
2011	118 (67)	59 (33)	70 (64)	40 (36)

Similarly, among females, CSMRs for NCDs did not show any significant trends. For example, at the start of 2003, there were about 17 NCD deaths per 10,000 PYs. The CSMR for NCDs then peaks at 56 deaths per 10,000 PY in 2005, drops to 26.5 deaths per 10,000 PYs in 2009, and finally rises to 37 deaths per 10,000 PYs in 2011. The CSMR for group I did, however, show a general downward trend from 124 deaths per 10,000 PYs in 2003 to 51 deaths per 10,000 PYs in 2011. Unlike males, however, injuries had the lowest CSMRs over the study period but did not show any clear pattern in trends – varying from 4.5 deaths per 10,000 PYs in 2003, down to 2.3 per 10,000 PYs in 2005, and peaking at 14 per 10,000 PYs in 2011. The normalized Mann–Kendall statistics for group I, II, and III causes did not indicate any significant trends (*Z*_S_=1.77, 0.52, and 0.73, respectively).

**Fig. 2:**
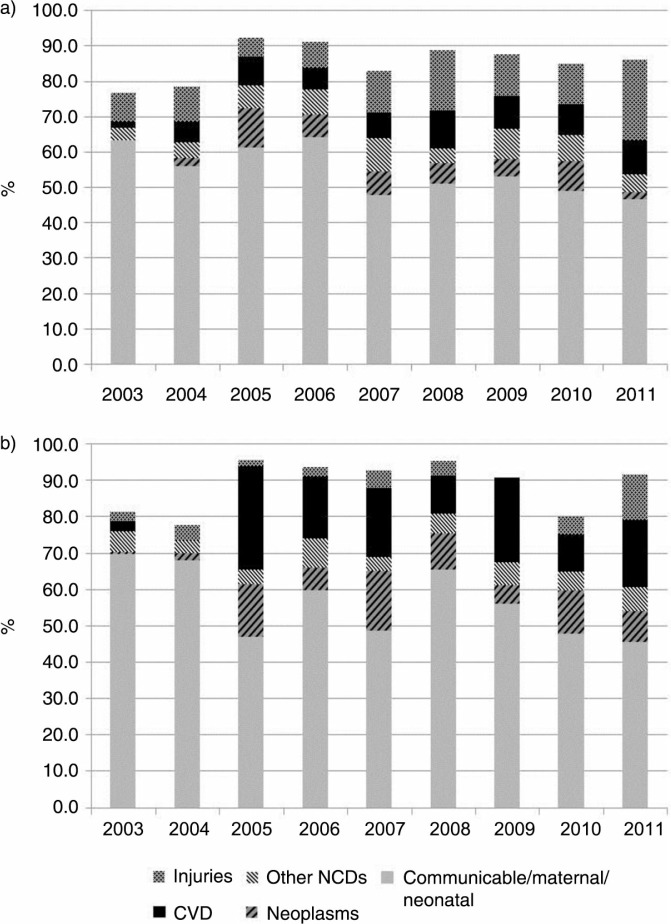
Trends in cause-specific mortality fractions among adults aged 35 years and older by year, 2003–2011: (a) males; and (b) females.

Table 2 shows the CSMFs for the broad groups of NCDs – neoplasms, CVDs, and other NCDs (see also Figure 2). Again, no clear patterns can be observed over time in CSMFs contributed by neoplasms, CVDs, or other NCDs among either males or females. What is clear is that, over time, group I conditions are consistently the most dominant contributor (54% on average) to mortality in both males and females. Among males, however, injuries are the next largest contributor – 12% on average over time. CVDs, neoplasms, and other NCDs contribute 8, 5, and 6%, respectively, of the CSMFs on average over time. Among females, CVDs are the second highest contributor to CSMF, accounting for 14% of mortality on average over time. This is followed by neoplasms (8%), other NCDs (5%), and injuries (4%).

**Table 2:** Cause-specific mortality fractions among adult males and females aged 35 years and older, 2003–2011

	Year of death								
	
	2003	2004	2005	2006	2007	2008	2009	2010	2011
Males									
Communicable, maternal, or neonatal	63.5	56.1	61.4	64.4	48.0	51.2	53.3	49.2	46.8
Neoplasms	0.0	2.4	11.0	6.3	6.7	5.7	4.9	8.4	1.8
Other NCDs	3.4	4.3	6.5	7.0	9.3	4.1	8.4	7.4	5.0
CVDs	1.7	5.9	8.3	6.1	7.4	11.0	9.4	8.8	9.8
Injuries	8.4	9.9	5.2	7.3	11.6	16.9	11.7	11.4	22.7
Indeterminate	23.0	21.5	7.6	8.9	17.1	11.1	12.3	14.8	13.8
Total	100.0	100.0	100.0	100.0	100.0	100.0	100.0	100.0	100.0
Females									
Communicable, maternal, or neonatal	69.7	68.1	47.2	59.8	48.9	65.6	56.2	47.9	45.8
Neoplasms	0.8	2.0	14.5	6.3	16.3	10.0	5.0	11.9	8.4
Other NCDs	5.5	3.5	3.9	8.1	3.7	5.3	6.3	5.3	6.6
CVDs	3.1	0.0	28.3	16.9	19.0	10.6	23.4	10.2	18.5
Injuries	2.5	4.2	1.9	2.7	5.0	3.9		4.7	12.6
Indeterminate	18.4	22.2	4.2	6.2	7.1	4.6	9.1	20.0	8.2
Total	100.0	100.0	100.0	100.0	100.0	100.0	100.0	100.0	100.0

NCD=non-communicable diseases; CVD=cardiovascular diseases.

## Discussion

We set out to demonstrate trends in NCD deaths among adults aged 35 years and older between 2003 and 2011 in the NUHDSS. The NUHDSS covers two urban slum settlements in Nairobi. As far as we know, no similar study exists that focuses on trends in the burden of NCDs in slums in Kenya or other parts of sub-Saharan Africa. Previous mortality studies in the slums of Kenya have looked at the burden of disease in general rather than in trends, or have focused on injuries (26, 43).

We did not find any clear trends in NCD mortality over time. However, we observed that, comparatively, trends in NCD mortality among males and females had remained stable over time. Group I conditions showed a declining trend, particularly among women. The declining trends in communicable disease, which constitute the bulk of Group I causes, mortality among adults are largely driven by reductions in AIDS and tuberculosis mortality over time (44). This most likely reflects the huge investments in HIV prevention, treatment, and control programs in Kenya and other developing countries over the past decade or so (45). Injuries did not show any significant changes over time, although males appeared to have a higher burden than females. This may be explained by the known fact that males tend to engage in high-risk behaviour such as violent crime, particularly in the slum context (26, 43).

Global projections show that NCD mortality will continue to increase in developing countries while communicable diseases will decrease. Concerns about these projections have led to unified global calls to action to combat NCDs, including the UN Summit on NCDs that was held in New York in September 2011 and the World Health Assembly Resolution on preventing and controlling NCDs in May 2012. However, rather than institute vertical programs to tackle the burden of NCDs, there have been calls for an integrated approach that aims at strengthening health systems in developing countries (13, 46). We believe that these calls are logical and relevant in our setting, where resources are scarce and need to be maximized. Moreover, we have evidence that certain risk factors for NCDs are on the rise in slum settings in Africa (7, 10). Therefore, the time to act is now.

Our study has a number of limitations. We could not explore age differentials due to the overall small numbers of NCD deaths. Also, again due to small numbers of NCD deaths, we could not meaningfully further disaggregate NCD deaths into more specific mortality causes beyond the three broad groups of neoplasms, CVDs, and other NCDs. Finally, the proportion of indeterminate causes contributed to between 4 and 23% of the CSMFs over time. Specifically, the years 2003 and 2004 yielded the highest proportion of indeterminate causes. It is difficult to explain this as InterVA-4 was applied uniformly to the data set across all years, and the similar data collection tools were used over the study period. However, this may be suggestive of data inconsistencies in the affected years.

## Conclusions

In conclusion, our findings are consistent with the recent Global Burden of Disease 2013 study which shows that Group I conditions remain the dominant cause of death in Africa, although NCDs and injuries still played a significant role over time (47). We recommend an integrated approach towards disease prevention that focuses on health systems strengthening in resource-limited settings such as ours.
